# Surgery of the Liver

**Published:** 1905-02-11

**Authors:** 


### SURGERY OF THE LIVER.

**Liver Abscess.**—Rhoads[1] in dealing with the diagnosis of hepatic abscess, says the features which characterise a typical case seen early in the course of the disease are as follows:—The patient gives a history of dysentery contracted in the tropics and has lost weight, his features are drawn, his complexion is ashy-brown, he suffers with languor, and complains of a dragging pain in his liver, his liver dulness is increased on percussion and has an area of tenderness, his temperature rises in the evening to 100° F. (pure amœbic type) or to 102° F. (mixed infection), the corresponding morning temperatures being 98° F. and 99° F.; his evening pulse is 95 (pure amœbic type) or 110 (mixed infection), the corresponding morning beats numbering 72 and 85. He has a leucocytosis of 12,500, 70 per cent. hæmoglobin, and 3,500,000 red blood-cells by count; the subcutaneous veins over the hepatic area are dilated; he has no jaundice or splenic enlargement; there are no friction sounds over the hepatic area, nor is there bulging of the chest wall, or local œdema; cough is not a symptom; basic pneumonia is not present, and there is no dyspnœa; the skin is moist; the tongue is coated with a greyish fur, and he is either constipated (post-dysenteric) or has an active chronic dysentery; his urine shows a trace of albumin, and at times casts; he feels chilly, but has no rigors; his brain is clear but inactive; he is generally an ambulatory case, but feels very much out of sorts, and is willing to resort to anything to be restored to health.

**Hepatic Cirrhosis.**—Deaver[2] says that hepatic cirrhosis may be the direct result of infection ascending the common duct from the duodenum, or through the portal circulation from the intestines. Several observers have shown that when infected bile is obstructed by ligature, the number of bacilli behind the point of ligature becomes enormously increased. Many writers believe that the retained bile, *per se*, does not occasion biliary cirrhosis, but that there must also occur inflammation of the bile ducts, cholangitis. In urging the possibility of biliary cirrhosis resulting from neglected gall-stones, the author says he is not unmindful of the fact that jaundice is absent in most cases, but there might be sufficient obstruction to lead to disease without there being sufficient to cause jaundice. In cases of hepatic cirrhosis, Talma[3] advocates suture of the omentum to the parietes in order to ensure adhesions, which become vascular, and thus relieve the portal system. It would be better if omentopexy were practised earlier in the course of cirrhosis. Alexandre deprecates operation in advanced cases. The omentum should be drawn down and spread out laterally, but it is important to avoid dragging on the transverse colon. The edge of the omentum is sutured to the parietal peritoneum on the right, the left, and the lower angle of the wound. Alexandre deprecates scratching the peritoneum, fixation of the edge of the omentum between the liver and diaphragm, and suture of omentum to the subcutaneous or submuscular tissue in front of the peritoneum. Occasionally hæmatemesis, and, in one case, hæmorrhage from the bowel, have followed omentopexy.

**Cholecystectomy.**—Lilienthal[4] is an advocate of primary removal of the gall-bladder in most cases of cholecystitis. He always drains through the wound after the duct is ligatured, having made sure of the patency of the common duct and lower end of the cystic duct. Roughly speaking, the cases may be divided into two classes, viz., those in which active infection is present and those in which there is no active infection. If there is no active infection, the matter of drainage of the bile ducts becomes unimportant, and one may as well remove the diseased organ which once has shown a vicious tendency. Yet, even in the second class, when active infection is present, it seems also wise to remove the septic viscus, and, if necessary, drain the biliary system, provided the anatomical conditions are not such as to make prolonged and difficult manipulation necessary. The types of gall-bladder disease which are not considered suitable for cholecystectomy are the following: (1) Extensive carcinoma with involvement of neighbouring viscera. (2) Cholecystitis with large perforations and pericholecystic abscesses, so that on entering the abscess with the finger one at the same time enters the gall-bladder. But even here the procedure will depend upon the extent of the involvement and the condition of the patient. (3) In cases of known hæmorrhagic tendency. (4) In obviously moribund patients. The author concludes as follows: (1) Cholecystectomy, while not absolutely insuring a cure of cholelithiasis, is the most radical procedure at our command. (2) The primary operation is far safer than the secondary. (3) Primary cholecystectomy is, on the whole, an operation at least as safe as appendicectomy. Moynihan[5] gives the following indications for the performance of cholecystectomy: (1) In injuries of the gall-bladder, rupture, stab or bullet wounds. (2) In gangrene of the gall-bladder. (3) In phlegmonous cholecystitis. (4) In membranous cholecystitis. (5) In chronic cholecystitis with dense thickening of the walls of the gall-bladder and cystic duct, with or without stenosis of the cystic duct, and in chronic cholecystitis when the gall-bladder is shrivelled and puckered and universally adherent. (6) In distension of the gall-bladder, hydrops or empyema, due to blockage of the cystic duct by calculus, stricture, growth, or external inflammatory deposits, or in cases of mucous fistula following operations for these conditions. (7) In cases of fistula between the gall-bladder or the cystic duct on the one hand, and the stomach, duodenum, or colon on the other. (8) In multiple ulcerations of the gall-bladder or the cystic duct when gall-stones have eroded their way through the walls into the liver, the duodenum or other protective adherent masses. (9) In primary carcinoma of the gall-bladder. Richardson[6] considers that cholecystectomy is almost, if not quite, as safe as cholecystostomy if done under similar conditions, and has the advantages of more rapid recovery and less liability to hernia. A contracted septic gall-bladder is best extirpated, but drainage must then be provided for the biliary passages. As gall-stones are formed in the gall-bladder, extirpation gives the best chance of preventing their re-formation. The one great argument for cholecystostomy is that good drainage of the biliary passages is thus obtained. Drainage is essential in inflammatory and septic cases, and is far safer through the gall-bladder than through a tube fixed in the hepatic duct. Cholecystectomy should therefore be the operation of last, not of first, resort.

Cholelithiasis.—Deaver[7] is convinced that operation should be resorted to as soon as it is definitely known that gall-stones are present. In general it may be said that the indications for medical treatment are the following:—(1) Acute obstructions of the common duct, for the stone having left the gall-bladder is moving through the common duct, and might reach the duodenum. (2) Mild attacks of colic, without any inflammatory symptoms, unless chronic invalidism is threatened. Such cases are markedly benefited by the Carlsbad treatment. (3) In cases of marked obesity, or on account of diabetes or disease of the heart, lung, kidney, or liver in which the shock of the operation would result fatally. (4) Carcinoma of the biliary apparatus, unless the obstruction required a palliative operation. In referring to the etiology of gall-stones, Clark[8] says that three facts have been prominently established:—(1) That the bile is not bactericidal. (2) That the micro-organisms in the gall-bladder are predisposing, if not absolute causative factors in the formation of gall-stones. (3) When gall-stones are present in the gall-bladder, infection in that viscus is much more likely to take place. He mentioned three avenues through which infection might enter the gall-bladder. (1) From bacteria circulating in the general blood-stream and reaching the liver through the hepatic veins. (2) By the direct passage of bacteria into the common bile duct through the duodenum. (3) By the transportation of bacteria from the intestine through the portal circulation. Knaggs[9] reports a case in which a gall-stone was removed from the gall-bladder, and in which there was subsequent development of malignant disease of the gall-bladder, and death from suppurative cholangitis.

[1] Annals of Surg., May 1904, p. 711. [2] Lancet, Aug. 13, 1904, p. 455. [3] Brit. Med. Jour., Sept. 17, 1904, p. 690. [4] Annals of Surg., July 1904, p. 44. [5] Lancet, April 30, 1904, p. 1190. [6] Med. Chron., July 1904, p. 271. [7] Lancet, Aug. 13, 1904, p. 456. [8] Med. Rec., July 16, 1904, p. 113. [9] Lancet, April 16, 1904, p. 1054.

